# The economic impact of Marfan syndrome: a non-experimental, retrospective, population-based matched cohort study

**DOI:** 10.1186/1750-1172-9-90

**Published:** 2014-06-23

**Authors:** Dmitrij Achelrod, Carl Rudolf Blankart, Roland Linder, Yskert von Kodolitsch, Tom Stargardt

**Affiliations:** 1 Hamburg Center for Health Economics (HCHE), Universität Hamburg, Esplanade 36, 20354 Hamburg, Germany; 2 WINEG – Scientific Institute of the Techniker Krankenkasse (TK) for Benefit and Efficiency in Health Care, Bramfelder Str. 140, 22305 Hamburg, Germany; 3 Department of Internal Medicine, Division of Cardiology, University Medical Center Hamburg-Eppendorf, Martinistraße 52, 20246 Hamburg, Germany

**Keywords:** Marfan syndrome, Economic impact, Cost-of-illness, Genetic matching, Administrative data

## Abstract

**Background:**

Marfan syndrome is a rare disease of the connective tissues, affecting multiple organ systems. Elevated morbidity and mortality in these patients raises the issue of costs for sickness funds and society. To date, there has been no study analysing the costs of Marfan syndrome from a sickness fund and societal perspective.

**Objective:**

To estimate excess health resource utilisation, direct (non-)medical and indirect costs attributable to Marfan syndrome from a healthcare payer and a societal perspective in Germany in 2008.

**Methods:**

A retrospective matched cohort study design is applied, using claims data. For isolating the causal effect of Marfan syndrome on excess costs, a genetic matching algorithm was used to reduce differences in observable characteristics between Marfan syndrome patients and the control group. 892 patients diagnosed with Marfan syndrome (ICD-10 Q87.4) were matched from a pool of 26,645 control individuals. After matching, we compared health resource utilisation and costs.

**Results:**

From the sickness fund perspective, an average Marfan syndrome patient generates excess annual costs of €2496 compared with a control individual. From the societal perspective, excess annual costs amount to €15,728. For the sickness fund, the strongest cost drivers are inpatient treatment and care by non-physicians. From the sickness fund perspective, the third (25–41 years) and first (0–16 years) age quartiles reveal the greatest surplus in total costs. Marfan syndrome patients have 39% more physician contacts, a 153% longer average length of hospital stay, 119% more inpatient stays, 33% more prescriptions, 236% more medical imaging and 20% higher average prescription costs than control individuals. Depending on the prevalence, the economic impact from the sickness fund perspective ranges between €24.0 million and €61.4 million, whereas the societal economic impact extends from €151.3 million to €386.9 million.

**Conclusions:**

Relative to its low frequency, Marfan syndrome requires high healthcare expenditure. Not only the high costs of Marfan syndrome but also its burden on patients’ lives call for more awareness from policy-makers, physicians and clinical researchers. Consequently, the diagnosis and treatment of Marfan syndrome should begin as soon as possible in order to prevent disease complications, early mortality and substantial healthcare expenditure.

## Background

Marfan syndrome is a rare, multisystem disease of the connective tissue, affecting multiple organ systems [1]. The prevalence is estimated at 1–3/10,000 in both male and female individuals [2]. Although most Marfan syndrome patients inherit the disease through an autosomal dominant genetic pathway within the family, 20–30% of new Marfan syndrome incidents are caused by de novo mutations in individuals with a previously unaffected family history [3,4]. Pathogenic mutations of the fibrillin-1 (*FBN1*) gene can cause dysregulation of transforming growth factor-beta 1 (TGF-b1) signalling, which is a polypeptide growth factor playing an important role in maintaining the integrity of the extracellular matrix. Consequently, excessive TGF-b1 causes a weakening of the tissues [5,6].

Marfan syndrome primarily manifests itself through skeletal and ocular malformations, diseases of the skin and of the neurological system as well as lung and cardiovascular conditions [7,8]. Mitral valve prolapse and aneurysmal disease of the aorta can be diagnosed in up to 90% [9-11] and 85% [9,12,13] of Marfan syndrome patients respectively. Aortic root dilation predisposes to severe chronic aortic regurgitation, aortic dissection and rupture [14]. Most systemic manifestations of the Marfan syndrome, especially cardiovascular ones, may appear in young individuals and are aggravated with increasing age as they are frequently the result of weakness in the connective tissue [15-17].

Thanks to the advancement in diagnostic techniques, such as non-invasive imaging technology and molecular genetic diagnostics [8,18], as well as refined surgical techniques, such as the remodelling of the aortic root [19-22], Marfan syndrome can be diagnosed and treated at an early stage. These developments have contributed to rising life expectancy from 30 to 60 years within the last three decades [23-25]. However, the high cardiovascular risk may require life-long prophylactic medication with β-blockers [24,26], angiotensin-converting enzyme inhibitors (ACE inhibitors) [5,8] or angiotensin receptor blockers (ARB) [7,27], as well as echocardiographic check-ups [28] and, if necessary, mitral valve replacement/remodelling [29,30] and aortic root surgery [31]. Permanent skeletal and ocular surveillance is also mandated [4,32,33].

Owing to increased Marfan syndrome-induced morbidity and mortality, Marfan syndrome patients require intensified utilisation of healthcare resources. Except for one study analysing one specific type of reimbursement of Marfan syndrome treatment in an outpatient clinic [34], there is no further evidence on the economic impact of Marfan syndrome. The authors are not aware of any nationwide study that analyses resource use across all healthcare sectors. Such studies, however, are crucial in shaping public health policy debates as they represent a valid economic basis for decision-makers, raise awareness and estimate the extent to which a disease has an impact on (a part of) society. They are regarded as an important tool in assisting policy planning, resource allocation, priority setting and can offer a basis for cost-effectiveness analyses of healthcare interventions [35]. High disease costs might suggest, for instance, that more emphasis should be placed on prevention and curative interventions at an early stage of disease progression. Consequently, the aim of this paper is to comprehensively and reliably estimate health resource utilisation, direct (non-)medical and indirect costs attributable to Marfan syndrome from a sickness fund and a societal perspective in Germany.

## Methods

### Study design and study sample

In order to estimate the economic impact of the Marfan syndrome, a non-experimental, retrospective, population-based matched cohort study design was applied. Costs for Marfan syndrome patients were compared with those of a matched cohort of patients not affected by the disease. The analysis is based on administrative claims data from Techniker Krankenkasse, Germany’s second largest sickness fund (out of 221 sickness funds) covering about 9% of the German population in 2008, i.e. 7.6 million insurees. The dataset included longitudinal patient-level information on socio-demographic status, direct (non-)medical costs as well as on healthcare utilisation between 2006 and 2008, such as employment status, costs for outpatient/inpatient treatment or physician contacts.

We used a bottom-up, prevalence-based approach for cost estimation. For the Marfan syndrome group, patients were required to have one inpatient ICD-10-GM (German modification) Q87.4 diagnosis or at least two outpatient Q87.4 diagnoses within 180 consecutive days in 2006–2008 [36]. The control group was built by randomly selecting up to 150 male and 150 female non-Marfan syndrome insurees per each year of age (0 to 100 years) from the same database, i.e. 26,645 people.

Outcomes were measured in the observation period between 1 January 2008 and 31 December 2008. In order to allow for risk adjustment, a period of 365 days (1 January 2007 to 31 December 2007) prior to the index date (1 January 2008) was stipulated as the basis for determining patient-level risk profiles. For each patient, an ICD diagnosis was included in their risk adjustment if it was made at least once in inpatient settings or at least twice within 180 consecutive days in outpatient settings. Hence, applying equally to the case and control groups, individuals were excluded from the study if they had not been constantly enrolled during the risk adjustment and observation period. Patients who died during this time frame are exempted from this imperative. Data mining and cost analysis were conducted with *SAS 9.3* (SAS Institute Inc.) and *R* software respectively [37,38].

### Study outcomes

Direct medical, direct non-medical and indirect costs were estimated from the sickness fund and the societal perspective. In order to achieve maximally transparent and comparable results, costs were structured following national standards into three distinct cost categories, ‘direct medical’, ‘direct non-medical’ and ‘indirect costs’ (see Table 1) [39].

**Table 1:** Cost categories from sickness fund and societal perspectives

**Cost type**	**Perspective**	
	**Sickness fund**	**Societal**
**Direct medical costs**		
Inpatient treatment	✓| ✓	✓| ✓^a^
Outpatient treatment	✓| ✓	✓| ✓^a^
Care by non-physicians	✓| ✓	✓| ✓
Pharmaceuticals	✓| ✓	✓| ✓^a^
Devices and medical appliances	✓| ✓	✓| ✓
Rehabilitation	✓| ✓	✓| ✓
Medical services (nursing care at home)	✓| ✓	✓| ✓
**Direct non-medical costs**		
Administration	✓| ✓	✓| ✓
Sick leave compensation	✓| ✓	✗| ✗
Travel expenses	✓| ✓	✓| Ⓧ^b^
Other non-medical services	✓| ✓	✓| Ⓧ^b^
Informal care by family caregivers	✗| ✗	✓| ✓
Patient time (loss of leisure time)	✗| ✗	✓| ✗
**Indirect costs**		
Reduced productivity at work	✗| ✗	✓| ✗
Lost production (absence, disability, premature death)	✗| ✗	✓| ✓

The first sign reflects the relevance of the cost item from the respective perspective, whereas the second sign reflects whether the item is included in this analysis.

✓relevant | included; ✗ not relevant | not included; Ⓧ partially included.

^a^comprises patient co-payments in addition; ^b^only comprises costs borne by sickness fund.

### Direct medical costs

First, direct medical costs are costs for inpatient/outpatient treatments, care by non-physicians (e.g. physiotherapy), pharmaceuticals, devices/medical appliances (e.g. prostheses), rehabilitation and medical services (e.g. home-based nursing care). Since the introduction of §116b Social Code Book (SGB) V in 2006, accredited hospitals can receive higher reimbursement prices for treating Marfan syndrome patients in an outpatient setting. Costs resulting from this funding scheme are described separately. To adjust direct medical costs for the societal perspective, co-payments for adult patients were added for outpatient consultations, pharmaceuticals, medical appliances, hospital stays and rehabilitations. According to the legislation, total annual co-payments were not allowed to exceed 1% of annual gross income for chronically ill people and 2% of income for all residual insurees. We used average wages in 2008 (men: €39,528; women: €31,932) [40] to calculate sex-adjusted co-payment thresholds according to §61 SGB V. The value added tax (VAT) of 19% applicable to pharmaceuticals and medical appliances was excluded in the societal perspective. Adding investment costs to the tune of 4.7% [41] of inpatient treatment costs was necessary from the societal perspective to reflect the fact that hospital investments are tax funded.

### Direct non-medical costs

Second, direct non-medical costs from the sickness fund perspective comprise administration costs, sick leave compensation paid by the sickness fund, travel expenses for physician appointments and ambulance transport as well as other non-medical services (e.g. housekeeping). Administration costs of €192 per insuree, based on the average administration costs of all German sickness funds, were applied [42].

Owing to the severe presentation of Marfan syndrome, affecting not only patients but probably also their relatives/partners [43], the cost of informal care by family caregivers was included in the societal perspective. For Marfan syndrome individuals under the age of 18 years, we assumed that at least one family member was caring for the person. Marfan syndrome patients over the age of 18 years were considered to receive informal care only if they were married or living with a partner (69.1%) [44]. We did not incorporate care received from friends or other relatives. As the number of hours per day that carers spend with their Marfan syndrome-inflicted family member/partner is unknown, we used literature on family care for patients with heart diseases. Because of the severe cardiovascular disease of Marfan syndrome patients, we assumed that these patients require approximately the same amount of informal care as patients with a heart disease (8 hours per day, SD: 4.1 hours) [45]. In order to estimate the costs of informal care in the control group, we assumed that the multisystemic nature of Marfan syndrome, usually affecting more than two organs/parts of the body, is comparable to a health state of an individual with at least two distinct comorbidities. Control subjects who fulfilled the criteria for at least two Elixhauser comorbidity groups (measured by one binary variable per comorbidity class) [46] were presumed to require the same amount of care hours per day as Marfan syndrome patients. Subsequently, the carers’ hours spent on informal care, for Marfan syndrome patients as well as for the control group, were valued at the average annual gross wage in 2008 (€37,236) [40].

Sick leave compensations were excluded from the societal perspective as they denote a transfer payment.

### Indirect costs

Being relevant from the societal perspective only, indirect costs consist of the cost of absence from work, the cost of premature death and the cost of reduced work productivity. The last factor could not be incorporated because our administrative data do not contain information about fluctuations in productivity. As recommended and widely applied [39,47], patient-level indirect costs were measured using the human capital approach in the baseline scenario. In a secondary analysis, we used the friction cost model (see Table 2) [48].

**Table 2:** Overview of two approaches for measuring indirect costs

	**Human capital approach (baseline analysis)**	**Friction cost approach (sensitivity analysis)**
**Perspective**	• considers *patient*’s hours of work that are lost (possibly until patient’s retirement age)	• considers patient’s hours of work that are lost until *employer* can replace the patient
**Lost production**	• measured through patient’s lost earnings (valued at 100%) no cap on duration of absence from work	• measured through patient’s lost earnings (valued at 80%) absence from work capped at 76 days (friction period)
**Premature death**	• value of lost hours of work (=earnings) that would have accumulated until patient’s retirement age	• value of lost hours of work (=earnings) + value of lost hours of work for a full friction period (valued at 100%)

The human capital approach measures production losses in terms of lost earnings: in our case, the number of sick leave days was multiplied by the average, sex-adjusted gross annual wage including employer contributions to social insurance in 2008 (men: €44,748; women: €36,144) [40]. Adjustments for workforce participation (proportion of a population in the active labour force) and employment rates were made. In the case of premature death, production losses were accumulated until the average retirement age of 65 years, adjusted for age- and gender-specific workforce participation/unemployment rates [49], survival probabilities [50], productivity growth (0.5%) [51] and discounting (3%) [52].

In contrast, the friction cost approach accounts for costs resulting from absence from work or premature mortality only during the time it takes to replace the missing worker. The time period until the worker is replaced is called the ‘friction period’ [48]. During the friction period itself (in Germany, 76 days [53]), the price of labour is not valued at 100% because internal workers can partially replace the missing individual and he/she can make up for the lost production on returning to work. Consequently, lost production was valued at 80% [48] when the absence from work was shorter than the average German friction period of 76 days [53]. If the absence from work was longer than this, productivity losses (at full value) were limited to 76 days, assuming that the worker had been completely replaced afterwards and the worker was not able to compensate for the forgone production. Premature death was treated as a fully valued productivity loss of a complete friction period.

In general, all (non-)medical and (in-)direct costs were winsorised (outliers are replaced by a specific percentile value) at their lowest 1st and highest 99th percentiles at cost category level in order to limit the distorting impact of outliers.

### Healthcare utilisation

Indicators for healthcare resource utilisation were constructed, including physician contacts, length of hospital stays (LOS) and number of hospital stays, number and average cost of prescriptions, number of magnetic resonance (MRT)/computerised tomographies (CT) as well as number of sick leave days.

### Subgroup analysis and economic impact of Marfan syndrome

Because disease manifestations and severity may progress throughout a patient’s lifetime, costs may differ throughout the lifetime. To obtain a more granulated picture of the cost distribution, the analysis was stratified into age quartiles of the examined Marfan syndrome population (I: 0–16 years, II: 17–24 years, III: 25–41 years, IV: >41 years).

We estimated the economic impact by multiplying the excess costs of Marfan syndrome by the number of diseased people in Germany. In addition to using prevalence rates obtained from our data, we used prevalence rates from the literature. According to international estimations, prevalence rates range from 1 [54] to 3 [2] per 10,000.

### Statistical analysis

The aim of this paper is to isolate the specific effect of Marfan syndrome on costs – the so-called ‘average treatment effect on the treated’ (ATT) [55]. However, observational studies may be subject to selection bias and to unbalanced population baseline characteristics because of a lack of randomisation. Thus, in order to reduce confounding due to unbalanced baseline characteristics between the Marfan syndrome patients and the control group as well as to determine the excess costs (ATT) of Marfan syndrome, ‘genetic matching’ (GM) was applied. GM is a multivariate algorithm that matches Marfan syndrome patients with their most similar control subjects on a set of observed covariates (e.g. age, sex, comorbidity). GM significantly improves the comparability of group baseline characteristics [56]. After GM, the difference in costs between the Marfan syndrome patients and the control group, i.e. excess costs, represents the effect specifically attributable to Marfan syndrome (ATT) [57].

Prior to applying GM, it is necessary to specify the covariates to match on. To minimise bias, we used a list of pre-specified variables that are considered to possess a high prognostic potential for the outcome (cost). Evidence suggests that gender, age [58], comorbidities [46] and pharmacy-based metrics (PBM) [59] are robust predictors of healthcare costs. Consequently, in the matching procedure, the covariates were sex, age, 29 of the total 31 Elixhauser comorbidity groups [46,60] and 30 of the total 32 PBM groups [59]. We excluded two Elixhauser comorbidity groups (‘mitral valve disease’, ‘aneurysms’) and two PBM groups (‘blockers/calcium channel blockers’, ‘ACE inhibitors/angiotensin II antagonists’) which are directly related to Marfan syndrome. All covariates were determined in 2007.

**Figure 1:**
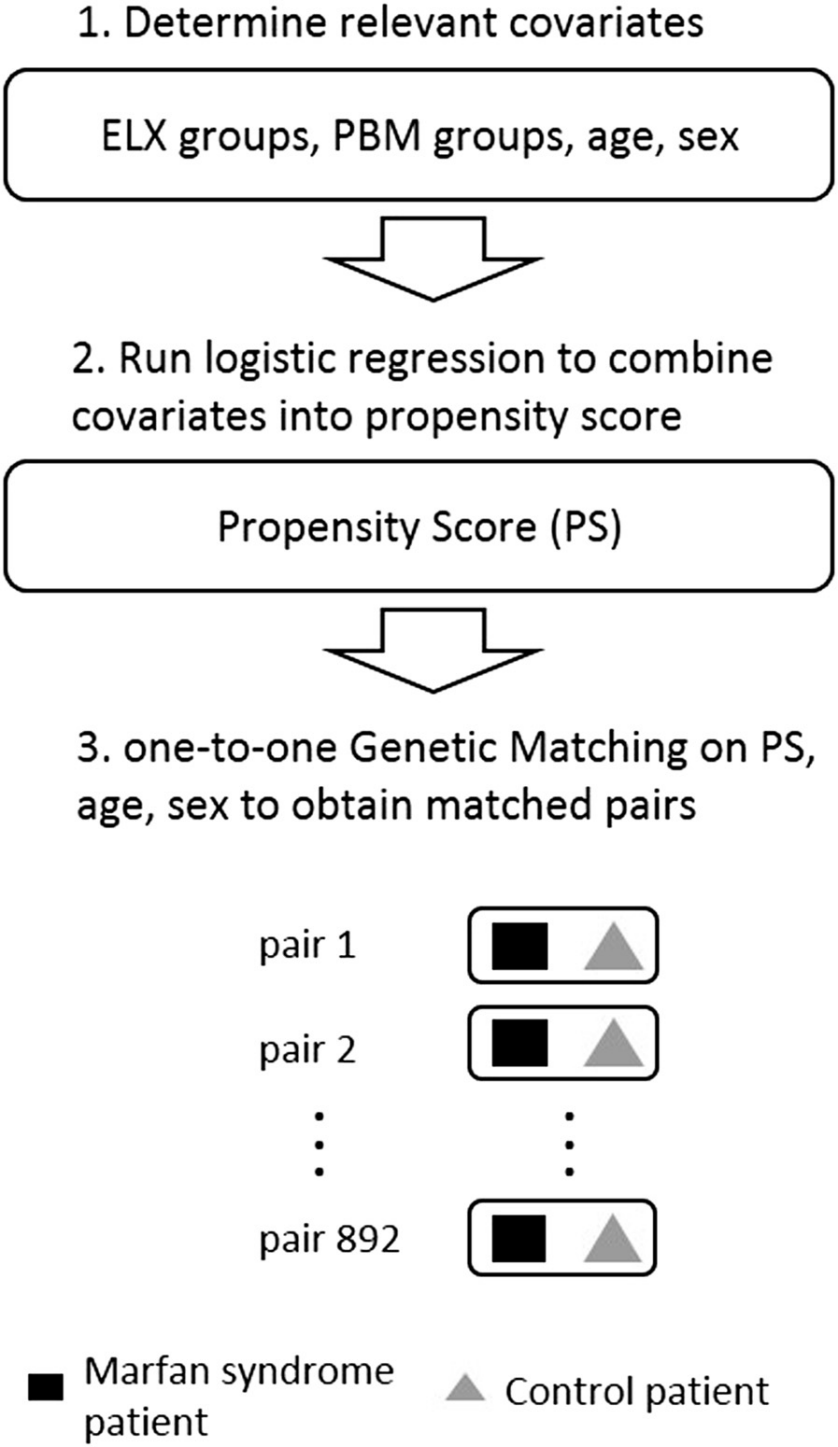
Overview of the steps in the statistical analysis/matching procedure.

First, using a logistic regression, a propensity score (PS) [61] was obtained by combining the above-mentioned covariates (sex, age, 29 Elixhauser comorbidity groups and 30 PBM groups). Subsequently, a one-to-one GM was run to attain balance on the PS, age and sex (see Figure 1). We applied GM with replacement as it will generally achieve the best balance on covariates and the smallest conditional bias [57]. In order to compare the balance of covariates before and after matching, bootstrap Kolmogorov–Smirnov (KS) tests and empirical quantile–quantile (eQQ) statistics were calculated [62].

### Sensitivity and secondary analyses

The statistical significance (p-values) of the matching results is only valid under the assumption that there are no unobserved confounders. Hence, Rosenbaum bounds were computed to estimate the impact of potentially existing hidden bias from unobserved covariates [63,64]. Rosenbaum bounds give an indication of how strong a hidden bias (*Γ*) must be such that it changes the inference about the causal effects from the matching procedure [64]. In sum, a model is insensitive to hidden bias if its conclusions change for large values of *Γ*, whereas it is sensitive if its conclusions alter for values that are only slightly larger than *Γ* =1.

To compare the baseline results with other methodological and structural specifications, three further analyses were conducted. First, we applied a pure PS model with the same parameterisations, but without the GM algorithm [65]. Second, the friction cost approach for estimating indirect costs was applied. Third, we carried out a sensitivity analysis on the lower-bound amount of hours for informal family care by subtracting one standard deviation from the mean (8–4.1 = 3.9 h/day).

## Results

In total, 892 individuals with Marfan syndrome and 26,645 control subjects were included. The prevalence of Marfan syndrome was 1.17 per 10,000 individuals within the population of Techniker Krankenkasse. These 892 Marfan syndrome patients were matched one-to-one with 892 control individuals. Overall, the GM drastically reduced differences in all baseline covariates (see Table 3). The mean age of the control group was reduced from 50.50 years to 28.90 years, removing statistical difference to the Marfan syndrome group (28.95 years). Similarly, the predominance of males was diminished to 40.70% in order to adjust to the Marfan syndrome group. The difference in the PS between the groups was no longer statistically different from zero (p = 0.999) after GM. Although the two groups had statistically significant differences (at p < 0.05) prior to matching in 15 of 29 Elixhauser groups, and in 16 of 30 PBM groups, the GM has removed all these divergences and has created a highly balanced distribution of clinical baseline characteristics (see Table 3 and Additional file 1: Table A1).

**Table 3:** Baseline characteristics of the Marfan syndrome patients and control group and balancing tests pre and post genetic matching

**Variables**	**Marfan**	**Control**	^**a**^**D-statistic**	^**b**^**p-value**
Sample size (N)				
before matching	892	26,645	–	–
after matching	892	892	–	–
Mean age (years)				
before matching	28.95	50.50	0.389	<0.001
after matching	28.95	28.90	0.002	0.996
% female				
before matching	40.70	50.45	–	<0.001
after matching	40.70	40.70	–	1
Mean propensity score				
before matching	0.116	0.029	0.470	0.002
after matching	0.116	0.116	0.002	0.999
Elixhauser comorbidities				
before matching	15 of 29 significantly different at p < 0.05			
after matching	0 of 29 significantly different at p < 0.05			
Pharmacy-based classes				
before matching	16 of 30 significantly different at p < 0.05			
after matching	0 of 30 significantly different at p < 0.05			

^a^D-statistic represents the maximum difference in the empirical cumulative distribution function (eQQ statistic).

^b^p-value: paired t-test for dichotomous and KS test for continuous variables.

### Sickness fund perspective

In both cost study perspectives, Marfan syndrome patients entail statistically significant higher costs (see Table 4). From the sickness fund perspective, an average Marfan syndrome patient generates higher direct medical costs (€2330, p < 0.001) and greater direct non-medical costs (€167, p < 0.001) than an average non-Marfan syndrome patient, resulting in total excess expenditure of €2496 (p < 0.001). Inpatient treatment (38.4%), care by non-physicians (33.8%), outpatient visits (10.7%) and pharmaceuticals (5.8%) constitute the greatest share of the total difference. In our data, 28 patients were treated within the reimbursement framework of §116b SGB V, which resulted in average costs of €2954 per patient per year. In total, the costs of medical services reimbursed through §116b as a share of the cost of all hospital treatments was 6.9%. Sick leave compensations represent the largest cost block in the direct non-medical costs with an excess of €143 (p < 0.001). These costs are directly related to the increased number of sick leave days taken by Marfan syndrome patients (9.23 vs. 5.02, p < 0.001).

**Table 4:** Average treatment effects for the treated (excess costs) in € (per capita, in 2008)

	**Sickness fund perspective**					**Societal perspective**				
	**Marfan**	**Control**	**ATT**^**a**^		**AI SE**^**b**^	**Marfan**	**Control**	**ATT**^**a**^		**AI SE**^**b**^
**Direct medical costs**	**4024**	**1695**	**2330**	***	**273**	**4105**	**1739**	**2366**	***	**278**
Outpatient treatment	780	512	268	***	35	800	531	269	***	35
Pharmaceuticals	385	241	145	**	46	349	222	127	**	39
Care by non-physicians	1315	472	843	***	163	1353	501	851	***	165
Devices and medical appliances	142	76	66	***	18	122	65	57	***	15
Inpatient treatment	1337	379	958	***	164	1413	403	1010	***	173
Rehabilitation	66	14	52	***	15	67	14	53	***	16
Medical services	<1	1	−1		5	<1	1	−1		2
**Direct non-medical costs**	**392**	**226**	**167**	***	**36**	**7431**	**1556**	**5875**	***	**116**
Administration	192	192	0		0	192	192	0		0
Sick leave compensation	161	18	143	***	34	n/a	n/a	n/a		n/a
Travel expenses	39	15	24	***	7	39	15	24	**	9
Other non-medical services	<1	<1	<1		<1	<1	<1	<1		<1
Informal family care	n/a	n/a	n/a		n/a	7200	1349	5851	***	116
**Indirect costs**	**n/a**	**n/a**	**n/a**		**n/a**	**10,329**	**2842**	**7487**	***	**718**
**Total costs**	**4416**	**1920**	**2496**	***	**286**	**21,865**	**6137**	**15,728**	***	**824.4**
**Indicators for healthcare utilisation**										
Sick leave days	9.23	5.02	4.21	***	1.11					
Physician contacts	10.11	7.28	2.83	***	0.26					
Average length of stay	3.06	1.21	1.85	***	0.52					
Inpatient stays	0.39	0.18	0.21	***	0.04					
Prescriptions	8.70	6.52	2.18	***	0.49					
Average prescription cost	27.58	23.04	4.55		3.61					
MRT/CT imaging	0.37	0.11	0.26	***	0.05					

^a^average treatment effect for the treated represents excess resource utilisation attributable to Marfan.

^b^Abadie–Imbens standard errors take the uncertainty of the matching process into consideration [57].

**< 0.01 ***< 0.001.

### Societal perspective

From the societal perspective, Marfan syndrome patients generate excess costs to the tune of €15,728 (p < 0.001). The surplus in direct medical costs (€2366, p < 0.001) differs only slightly from the sickness fund perspective. However, excess direct non-medical costs are substantially higher at €5875 (p < 0.001) and represent 37.4% of the total excess costs. This is primarily caused by the introduction of costs for informal care by family caregivers (€5851, p < 0.001), which are considerably higher for the Marfan syndrome group (€7200) than for the control group (€1349). In addition, indirect costs for lost production come into play with a surplus of €7487 (p < 0.001).

### Healthcare utilisation

In terms of healthcare utilisation, Marfan syndrome patients had 38.8% more physician contacts (p < 0.001), a 153.3% longer average length of stay if hospitalised (p < 0.001), 119.0% more inpatient stays (p < 0.001), 33.4% more prescriptions (p < 0.001), 236.3% more MRTs/CTs (p < 0.001) and 19.7% higher average prescription costs than control individuals (see Table 4).

### Subgroup analysis

**Figure 2:**
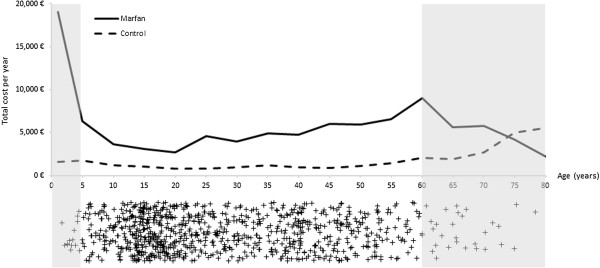
Total costs per capita for Marfan syndrome patients and control individuals, from the sickness fund perspective.

Total costs per year of age from the sickness fund perspective are depicted in Figure 2. Because the frequency of Marfan syndrome patients in our study group below the age of 5 years and above the age of 60 years is low, as represented by the shaded area in the scatterplot, reliable estimates can be drawn only for age quartiles or for the population between the ages of 5 and 60 years. For instance, the great cost difference in children up to the age of 5 years results from three outliers that incurred exceptionally high costs for physiotherapy. The third age quartile (25–41 years) reveals the greatest surplus in total costs (€2711), followed by the first (0–16 years, €2322) and fourth (>41 years, €2209) quartiles (see Additional file 1: Table A2). For quartile III, the main excess cost drivers are direct medical costs (€2394) including inpatient treatment (€1434), care by non-physicians (€492) and outpatient visits (€282). In the lowest age quartile (0–16 years), care by non-physicians (€1061), inpatient (€811) and outpatient treatments (€222) are the major cost blocks of excess direct medical costs (€2311). From the societal perspective, the second (17–24 years: €27,636) and third quartiles (€15,146) cause the greatest excess expenditure. This is mainly a result of the high costs of lost production due to premature death in young to middle-aged individuals. Excess costs of informal care by family caregivers are highest in the youngest group (QI: €9121) because children receive more family care than middle-aged individuals according to our model specifications. However, no production losses accrue to children.

### Economic impact of Marfan syndrome

Given a German population of 82.002 million in 2008 and depending on the prevalence rates (1.17–3/10,000), 9600 to 24,600 individuals are expected to have Marfan syndrome. Applying the cost data from our study, the overall cost of disease in 2008 from the sickness fund perspective ranged between €24.0 and €61.4 million, whereas the societal cost extended from €151.4 to €386.9 million.

### Results from sensitivity analysis

The Rosenbaum bounds test reveals that our results are highly robust to potentially existing unobserved confounders (see Additional file 1: Table A3). The lower and upper p-value bounds show whether unobserved confounders of the magnitude *Γ* have an influence on our results. For most outcome parameters, even a very strong hidden bias of the magnitude *Γ* =3 has no influence on the inference of the matching (at p < 0.05). Concerning pharmaceuticals, for instance, at a magnitude of bias of *Γ* = 2.5, one would not be able to reject the hypothesis (pU = 1) that unobserved covariates have an impact on the conclusions of the matching. Hence, at *Γ* = 2.5, the excess drug costs might not be a causal effect of Marfan syndrome but possibly result from unobserved confounders.

Matching with a PS model instead of GM did not lead to substantially different results, showing that our model is robust to different specifications of the matching algorithm. As anticipated, replacing the human capital approach with the friction cost model to value the costs of lost production considerably reduced the excess indirect costs to €359 (−95.26%), with highest excess values in the third age quartile (€999). Overall, the friction cost model yields an ATT of total costs of €8599, which is 44.24% lower than that in the baseline scenario (see Table 5).

**Table 5:** Sensitivity and secondary analyses with propensity score matching and friction cost model (values per capita in 2008)

	**Baseline (GM)**	**PS model**		**Friction cost model**	
	**ATT**^**a**^**(in €)**	**ATT**^**a**^**(in €)**	**% change**^**b**^	**ATT**^**a**^**(in €)**	**% change**^**b**^
**Sickness fund perspective**					
Direct medical costs	2330	2024	−13.14	2330	0
Direct non-medical costs	167	161	−3.58	167	0
Total costs	2496	2184	−12.49	2496	0
**Societal perspective**					
Direct medical costs	2366	2059	−12.97	2366	0
Direct non-medical costs	5875	5790	−1.44	5875	0
Indirect costs	7487	7573	1.15	359	−95.26
Total costs	15,728	15,422	−1.95	8599	−44.24

^a^average treatment costs of the treated (ATT) represent excess costs attributable to Marfan.

^b^percentage change compared with baseline GM model.

Decreasing the amount of daily hours of family caregiving to 3.9 h diminished Marfan syndrome patients’ excess costs for informal family care from €5875 to €2852 (p < 0.001). Consequently, the total societal cost difference amounted to €12,729 (p < 0.001) compared with €15,728 in the baseline scenario.

## Discussion

To the knowledge of the authors, this is the first study worldwide to representatively examine the economic impact attributable to Marfan syndrome across in/outpatient sectors from a sickness fund and societal perspective with claims data. The analysis used a dataset with a large sample of insurees and a detailed account of costs and health resource utilisation. The demographic structure of the insurees in this dataset is representative of the overall German population [66]. A powerful matching algorithm (‘genetic matching‘) was applied to balance observable confounders and to extract the causal effect of Marfan syndrome on costs. In contrast to other cost-of-illness studies, which simply total all medical or diagnosis-related costs without a comparison group, the reliability of our results is high because we identified the excess costs that are specifically attributable to Marfan syndrome [67].

From a sickness fund and societal perspective, the average excess expenditure per Marfan syndrome patient in 2008 was €2496 and €15,728 respectively. Costs mainly resulted from inpatient stays, care by non-physicians, outpatient treatments as well as informal family care and production losses in the societal perspective. Although Marfan syndrome patients required higher incremental drug expenditure (sickness fund: €145), the difference was not large because the most frequently applied pharmacological therapy for Marfan syndrome – beta-blockers [68] – is relatively inexpensive.

Cost differences between Marfan syndrome and control subjects were highest in the third (25–41 years) and first (0–16 years) age quartiles from the sickness fund perspective. These results agree with the characteristics and progressive development of the disease. On the one hand, childhood and adolescence are regarded as the decisive period for the development of cardiovascular diseases in Marfan syndrome [69]. Hence, costs for Marfan syndrome patients in their early years of life may most likely be elevated because their diagnosis needs to be established using costly methods including *FBN1* gene sequencing, regular cardiovascular check-ups, ophthalmological care [8] and potentially life-long pharmacological therapy [1]. Their high costs of care by non-physicians can be explained by the need to treat skeletal malfunctions, such as physiotherapy for scoliosis or pectus deformities [8,43,70]. In addition, costs in the youngest quartile might be driven by newborns with the severe manifestation of the disease or by the neonatal Marfan syndrome [33]. Most of these infants have a life expectancy of less than 1 year of age and are highly treatment intensive [71]. On the other hand, patients in the third age quartile have to cope with symptoms of Marfan syndrome that have been aggravated over time. Cardiovascular manifestations often do not become overt and diagnosed until the third decade of life [10]. By that time, progressive aortic dilatation and aneurysms often dictate aortic and mitral valve surgery [68,72]. As shown in a recent meta-analysis, the mean age of Marfan syndrome patients undergoing cardiac surgery lies somewhere in their early/mid-thirties [14]. Our data corroborate this finding, indicating that inpatient stays, mostly because of cardiovascular surgeries, constitute 59% (€1434) of the excess direct medical costs in the third age quartile.

Owing to the systemic nature of their disease, Marfan syndrome patients require significantly more healthcare resources than control subjects, such as prescriptions, physician visits and inpatient stays/duration. Depending on the prevalence rate, the total economic impact ranges between €24.0 and €61.4 million (sickness fund perspective) and €151.3 and €386.9 million (societal perspective). Owing to the high number of sick leave days, Marfan syndrome has a considerable impact on employability.

Putting this study into the context of the scientific literature is challenging because, so far, very little evidence exists on the cost of illness of Marfan syndrome. Unrestricted comparisons cannot usually be made across different cost-of-illness studies on account of diverging methodologies [67], such as total cost vs. incremental/excess cost studies. One German micro-costing study without a control group estimated that the average direct medical cost – measured by resource consumption – of treating a Marfan syndrome patient in their specialised outpatient clinic in 2008 amounted to €389 [34]. This figure is lower than our result (€780) because costs that accrued at other in/outpatient sites, such as expensive genetic testing, were not incorporated in that study [34]: in practice, however, the numerous morbidities of Marfan syndrome compel patients to see different in/outpatient specialist clinics, such as orthopaedics, ophthalmology and cardiology.

In terms of studies examining other rare diseases with a similar methodology, Blankart et al. (2013) [73] showed that costs attributable to chronic lymphocytic leukaemia (CLL) amounted to €4946 from a sickness fund perspective and €7910 from a societal perspective. In comparison with common chronic diseases, such as diabetes (€19,677 m in 2010) [74], the attributable societal costs of Marfan syndrome are relatively low (€386 m in 2008) because of its rare occurrence (1–3/10,000). Nonetheless, considering that the costs and prevalence of Marfan syndrome constitute a fraction of 1.97% of the costs and 0.34% of the prevalence of diabetes (890/10,000) [75], it becomes clear that the economic impact per Marfan syndrome patient is nearly six times higher than the impact per diabetes patient.

### Limitations

We see four limitations to our study. First, although administrative data provide a comprehensive picture of healthcare costs and are increasingly recognised in health services and epidemiological research [76,77], our data do not provide any information on the clinical progression and/or severity of disease. For instance, neonatal Marfan syndrome or specific mutations in the *FBN1* gene [12] may be more severe than other forms of Marfan syndrome, e.g. a manifestation of ectopia lentis and skeletal abnormalities. Consequently, healthcare resource utilisation and expenditure may vary according to disease severity. Costs at different developmental stages/severities of Marfan syndrome would be a factor worth considering in future research. Furthermore, our data do not include information about costs relating to public welfare budgets, such as costs for pension funds. Another pitfall of administrative data is the usage of the ICD catalogue, where some disorders similar to Marfan syndrome, such as the Shprintzen–Goldberg syndrome, may not be coded correctly because the current ICD classification includes only codes for about 500 out of the 8000 named rare diseases [78]. However, we believe that those ultra-rare diseases (estimated prevalence of Shprintzen–Goldberg syndrome is less than 50 cases in Europe [79]), which might be coded incorrectly, do not substantially bias our results.

Second, although the costs of genetic testing for *FBN1* mutations were included in the inpatient sector (through §116b reimbursement), it was not possible to account for them in the outpatient sector. Data coding modalities did not allow us to measure genetic testing costs for 20 Marfan syndrome patients. Such genetic testing, however, can be very expensive. Therefore, we might slightly underestimate outpatient treatment costs.

Third, we were not able to quantify intangible dimensions of indirect costs. For instance, Marfan syndrome patients face significant restrictions with respect to their quality of life, such as chronic pain [80] or psychological distress [44,81], which may in turn affect productivity [82,83]. Similarly, because of an absence of information on treatment schedules/time spent coping with Marfan syndrome, patients’ loss of leisure time was not included.

Finally, the inferences from the matching rely on the assumption that all relevant covariates have been included and that no unobserved confounders exist (‘unconfoundedness assumption’) [84]. This assumption is not empirically testable because hidden confounders cannot be measured. Nonetheless, one can assess the plausibility of the ‘unconfoundedness assumption’ by measuring the impact of the hypothetical existence of hidden confounders on the inferences, as we did using Rosenbaum bounds. Our results suggest that unconfoundedness is plausible because hidden bias (*Γ*) had little impact on outcomes.

## Conclusion

Relative to its low frequency, the treatment of Marfan syndrome requires high healthcare expenditure. Not only the high costs of Marfan syndrome but also its great burden on patients’ lives call for more awareness from policy-makers, physicians and clinical researchers. Early diagnosis and prophylactic treatment may limit cardiovascular complications in particular and thus contain healthcare expenditure and patient morbidity. Use of health information technology, such as patient medical records, and enhanced coordination among healthcare providers across sectors might improve the medical management of rare diseases [85,86]. Finally, knowledge about the pathogenesis of the disease is increasingly being gained, and it is expected that the accomplishments of translational research in molecular biology will soon open a window of opportunity for treating most systemic manifestations of Marfan syndrome [24].

## Competing interests

RL is employed by Techniker Krankenkasse, a large German sickness fund, and may have potential competing interests according to the guidelines of the International Committee of Medical Journal Editors. YK is the chairman of the non-profit patient advocacy organisation ‘Marfan Hilfe e.V.’. DA, CRB, YK and TS declare that they have no competing interests.

## Authors’ contributions

CRB and TS collected the data. RL participated in the coordination of the data collection. DA, CRB, and TS developed the design of the study and performed the statistical analysis. DA, CRB, RL, YK and TS drafted the manuscript. All authors read and approved the final manuscript.

## Supplementary Material

Additional file 1: Table A1 Elixhauser comorbidity groups and pharmacy-based metrics before and after matching with balance statistics. **Table A2.** Cost estimates in € for Marfan and controls, by age quartiles and analysis perspective (per capita in 2008). **Table A3.** Sensitivity analysis of estimated outcomes with Rosenbaum bounds at different magnitudes of hidden bias (1.0≤*Γ*≤3.0). Click here for file
